# Small-Molecule Detection in Biological Fluids: The Emerging Role of Potentiometric Biosensors

**DOI:** 10.3390/ijms262311604

**Published:** 2025-11-29

**Authors:** Nikola Lenar, Beata Paczosa-Bator

**Affiliations:** Faculty of Materials Science and Ceramics, AGH University of Krakow, Mickiewicza 30, PL-30059 Krakow, Poland

**Keywords:** potentiometric biosensors, small-molecule detection, biological fluids, point-of-care diagnostics, solid-contact electrodes

## Abstract

Detecting small molecules in biological fluids is essential for diagnosing diseases, monitoring therapy, and studying how the body works. Traditional biosensing methods—such as amperometric, optical, or piezoelectric systems—offer excellent sensitivity but often rely on complex instruments, additional reagents, or time-consuming sample preparation. Potentiometric biosensors, by contrast, provide a simpler, low-power, and label-free alternative that can operate directly in biological environments. This review explores the latest progress in potentiometric biosensing for small-molecule detection, focusing on new solid-contact materials and advanced sensing membranes and compact device designs. We also discuss key challenges, including biofouling, matrix effects, and signal drift, together with promising strategies such as antifouling coatings, nanostructured interfaces, and calibration-free operation. Finally, we highlight how combining potentiometric sensors with artificial intelligence, digital data processing, and flexible electronics is shaping the future of personalized and point-of-care diagnostics. By summarizing recent advances and identifying remaining barriers, this review aims to show why potentiometric biosensors are becoming a powerful and versatile platform for next-generation biomedical analysis.

## 1. Introduction

The detection of small molecules in biological fluids is a key aspect of modern diagnostics, providing critical information for metabolic monitoring, therapeutic drug monitoring (TDM), and the identification of disease biomarkers [1,2]. Conventional laboratory methods such as liquid chromatography coupled to mass spectrometry (LC–MS) or high-performance liquid chromatography (HPLC) deliver excellent sensitivity and selectivity but are constrained by high capital and operational costs, complex sample preparation, and the need for specialized personnel and centralized laboratories [3]. These limitations motivate development of portable, low-cost, and rapid sensing platforms suitable for point-of-care (POC) and near-patient testing.

Electrochemical approaches, particularly potentiometric sensors, have attracted interest because they offer simple instrumentation, fast response, low power consumption and straight miniaturization for POC applications [4,5]. Potentiometric biosensors detect changes in electrical potential at a sensing interface (typically a sensing electrode vs. a reference) and often rely on ion-selective membranes, molecularly imprinted polymers (MIPs), enzymes, aptamers or other recognition elements to ensure selectivity for target small molecules [4,6]. Recent reviews summarize advances in potentiometric transduction, solid-contact ion-selective electrodes (SC-ISEs), and the incorporation of nanomaterials and novel solid contacts to improve stability and limit of detection [4,5,7].

Applying potentiometric sensors to real biological matrices (blood, plasma/serum, urine, saliva, sweat) introduces specific analytical challenges: high ionic strength, protein adsorption (biofouling), matrix interferences, and drift/long-term stability of the reference and sensing interfaces [7,8]. Addressing these issues has prompted work on robust solid contacts, anti-fouling surface chemistries, integrated sample handling (microfluidics, filtration) and calibration strategies targeted at POC use [7]. Moreover, there is growing interest in potentiometric platforms for continuous or near-real-time monitoring (e.g., wearable/implantable formats) and for TDM applications, where rapid feedback on small-molecule concentrations could guide therapy [2,6].

**Figure 1:**
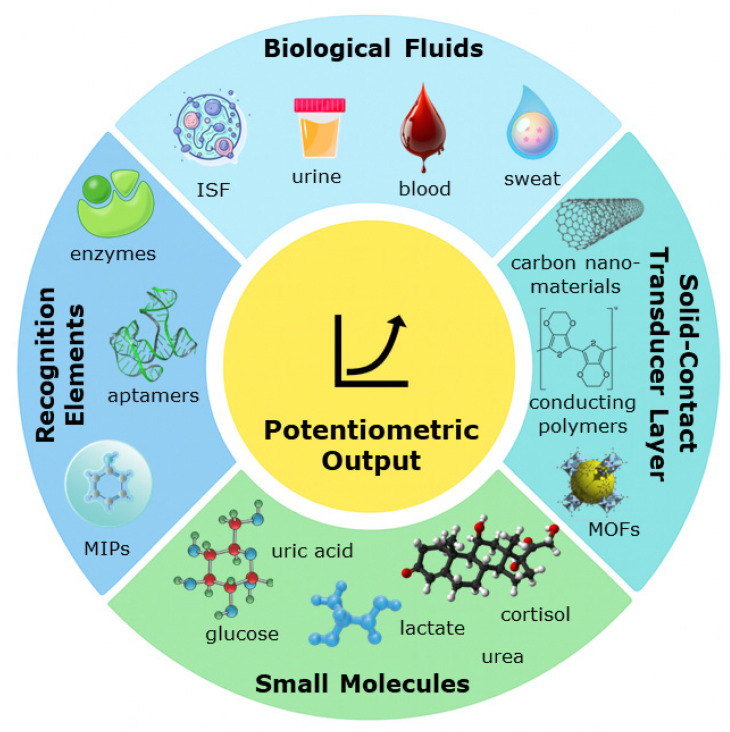
Visual summary of the overall objective and scope of the work (ISF—interstitial fluid; MOFs—Metal–Organic Frameworks; MIPs—Molecularly Imprinted Polymers).

Although several reviews have addressed potentiometric and electrochemical point-of-care (POC) sensors, few have examined in detail how potentiometric biosensors perform in detecting small molecules within biological fluids. The present work provides an updated overview of this area, emphasizing fundamental principles, recent advances in materials and recognition chemistries, analytical challenges specific to complex biofluids, and emerging directions toward real-time and clinical applications. Figure 1 provides an overview of the key structural layers and materials commonly used in potentiometric biosensors, highlighting how recognition elements (e.g., aptamers, MIPs, enzymes), membrane components, and solid-contact transducers integrate to generate a measurable potentiometric output in biological fluids such as blood, sweat, saliva, and interstitial fluid.

## 2. Fundamentals of Potentiometric Biosensing

Potentiometric biosensing involves the measurement of electrical potential differences between a sensing (indicator) electrode and a reference electrode under conditions of negligible current flow. The sensing electrode responds to biochemical recognition events, such as enzyme reactions, aptamer binding, molecular imprinting or antibody–antigen interaction, that lead to ionic or charge changes at the interface, which are transduced into a potential output [3,9,10,11]. In a simplified form, the Nernst Equation (1) governs the ideal behavior of ionic-activity-based sensors:

(1) $$E=E_{0}\pm \frac{RT}{zF}\ln\left(a_{ion}\right)$$

where (E) is the electrode potential, (E_0_) is standard electrode potential, (a_ion_) is the activity of the ion, (z) its charge, (F) is Faraday constant and (R) gas constant.

### 2.1. Principles and Recognition Agents

Classical ion-selective electrodes (ISEs) consist of a polymeric membrane (often PVC combined with a plasticizer and ionophore) and an internal filling solution, which presents limitations in size, maintenance, and suitability for miniaturization [12]. To overcome these, solid-contact ion-selective electrodes (SC-ISEs) have been developed: they replace the internal liquid electrolyte with a solid transducer layer (e.g., conductive polymers, carbon nanotubes, metal nanoparticles, ionic liquids), which acts as the ion-to-electron interface. This enables improved mechanical stability, reduced size, simpler packaging, and suitability for biosensor integration [13,14,15]. Recent work shows that nanocomposite solid contacts markedly improve stability and lower drift in potentiometric measurements [4].

When moving from purely ionic sensing toward the detection of small biomolecules (metabolites, drugs, and disease biomarkers), the biorecognition interface becomes the defining component of potentiometric biosensors. Bioreceptors such as enzymes, aptamers, antibodies, and molecularly imprinted polymers (MIPs) are integrated with the potentiometric transducer to ensure chemical selectivity and biological specificity.

In these systems, the recognition element—whether it involves enzymatic catalysis, specific binding, or molecular imprinting—alters the local ion activity, charge distribution, or interfacial potential, which is then transduced into a measurable potential shift. For instance, enzyme-based potentiometric sensors often exploit the generation or consumption of charged species (e.g., H^+^, NH_4_^+^, CO_2_) during substrate conversion, while aptamer- and antibody-based sensors rely on conformational or charge changes upon target binding that modulate the interfacial electric field.

A potentiometric sensor employing a dopamine-specific aptamer immobilized onto a detection membrane demonstrated that binding kinetics and equilibrium constants can be quantified directly from potential changes [1,16]. Similarly, molecularly imprinted polymers (MIPs) have emerged as robust synthetic alternatives to biological receptors. In solid-contact potentiometric sensors, MIPs tailored for neutral dopamine achieved sub-micromolar detection limits via reversible, non-covalent binding interactions [17]. Their chemical resilience, reusability, and compatibility with organic solvents make MIPs particularly attractive for long-term or in-field applications.

The composition of the membrane matrix plays a crucial role in the integration of biorecognition elements with the potentiometric transducer. While classical ISE membranes are primarily based on PVC and plasticizers, biosensing applications often require modified or alternative polymeric matrices that can immobilize biological or synthetic receptors without compromising ion transport. Enzyme-based potentiometric biosensors frequently employ PVC, polyurethane, or sol–gel membranes capable of enzyme entrapment, enabling localized generation of ionic products upon substrate recognition. In contrast, aptamer- and antibody-based sensors often rely on functionalized polymer surfaces or nanocomposite membranes incorporating carbon nanomaterials or metal nanoparticles to enhance surface area and electron transfer. The development of biocompatible hydrogel and sol–gel layers have further expanded membrane design, allowing stable receptor incorporation while maintaining the flexibility required for miniaturized, solid-contact biosensors [18].

Recent research emphasizes nanostructured and hybrid biorecognition architectures, where nanomaterials (e.g., gold nanoparticles, carbon nanotubes, graphene derivatives) serve dual roles, enhancing surface area for receptor immobilization and improving charge-transfer kinetics. The combination of biological selectivity with nanoscale transduction efficiency has enabled potentiometric biosensors capable of rapid, label-free, and low-power detection of small analytes in complex biological fluids.

### 2.2. Performance Parameters and Analytical Challenges

Key performance parameters in potentiometric biosensing include sensitivity (slope of the calibration curve, typically expressed in mV/decade), limit of detection (LOD), linear dynamic range, selectivity (often quantified through selectivity coefficients), potential drift (stability over time), and response time [19]. Sensitivity and LOD are directly influenced by the efficiency of ion-to-electron transduction and the quality of the biorecognition layer, while linearity defines the concentration range over which reliable quantification is possible.

Selectivity remains a major challenge in biosensing applications. Unlike traditional ion-selective electrodes that target single ionic species in simple matrices, potentiometric biosensors often operate in complex biological fluids such as serum, saliva, or sweat, where co-existing ions, metabolites, and macromolecules can interfere. Factors such as high ionic strength, variable ionic activities, and protein adsorption (biofouling) may disturb the potential response and obstruct accurate calibration [4,5].

Another persistent issue is electrode stability, which critically affects potential reproducibility, especially in miniaturized or wearable formats. Additionally, water-layer formation beneath polymeric or biorecognition membranes can cause signal drift and hysteresis. Miniaturization further introduces higher interfacial resistance and lower capacitance, amplifying electrical noise and potential instability [20].

To overcome these analytical limitations, recent work has focused on improving interfacial engineering and membrane design. The use of hydrophobic, nanostructured solid contacts and composite membranes helps suppress water-layer formation and enhance potential stability. Antifouling surface modifications (e.g., polyethylene glycol coatings, zwitterionic polymers) and biocompatible hydrogels reduce nonspecific adsorption in biofluids. Moreover, calibration-free and drift-compensated sensing schemes, including ratiometric and differential potentiometric configurations, are emerging as powerful strategies for reliable long-term monitoring in real biological environments.

Overall, maintaining stability, reproducibility, and selectivity under physiologically relevant conditions remains one of the key analytical challenges in translating potentiometric biosensors from laboratory prototypes to clinically applicable devices.

## 3. Target Analytes: Small Molecules in Biological Fluids

### 3.1. Classification of Small Molecules (Metabolites, Drugs, Biomarkers)

Small molecules, typically defined as organic compounds with molecular weights below approximately 1000 Da, represent a broad class of biologically and clinically relevant analytes. These include metabolites (e.g., uric acid, creatinine, glucose, lactate), therapeutic drugs (e.g., antibiotics, antiepileptics, statins, and analgesics), and biomarkers such as hormones, neurotransmitters, small peptides, and toxins [8]. The detection and quantification of these small-molecule species in biological fluids, such as blood, urine, saliva, and interstitial fluid, provide valuable insights into metabolic function, disease progression, and therapeutic efficacy [21].

In this context, small molecules can be broadly categorized according to their functional role in biological systems and analytical detectability:

(a) Metabolic indicators, such as glucose, lactate, or uric acid, often serve as substrates in enzyme-based potentiometric biosensors, where ionic products of enzymatic reactions generate measurable potential shifts.
(b) Pharmaceutical compounds, including antibiotics, anticancer drugs, and psychotropic agents, are typically detected through aptameric or MIP-based receptors that exploit molecular recognition and charge redistribution at the membrane interface.
(c) Endogenous or exogenous biomarkers, such as hormones, neurotransmitters, and environmental toxins, are increasingly being targeted using hybrid nanostructured recognition layers that combine molecular selectivity with enhanced signal stability.

Despite their clinical importance, small-molecule targets present distinctive analytical challenges. They often occur at low and variable concentrations, display structural and chemical similarity to endogenous species, and exist within highly complex biological matrices containing proteins, salts, and macromolecular components that can interfere with selective recognition [22]. Furthermore, many small molecules are uncharged or weakly ionic, complicating their direct detection through conventional potentiometric ion-selective electrodes.

Potentiometric biosensing approaches address these difficulties by coupling selective biorecognition elements (e.g., enzymes, aptamers, or molecularly imprinted polymers) with sensitive solid-contact transducers, enabling charge transduction even for neutral or weakly charged molecules. For instance, potentiometric sensing of dopamine using an aptamer-functionalized membrane demonstrated that small-molecule binding kinetics can be monitored through potential variations associated with conformational changes at the electrode interface [23].

### 3.2. Biological Fluids

Biological fluids serve as valuable non-invasive or minimally invasive matrices for monitoring small molecules, providing complementary information on physiological and pathological states. The choice of biological medium strongly influences sensor design, analytical performance, and calibration strategy, as each fluid presents distinct composition, ionic environment, and sampling feasibility.

Blood and plasma remain the gold standard for clinical diagnostics due to their direct reflection of systemic metabolism and drug levels. High protein content, ionic strength, and viscosity can cause electrode fouling, nonspecific adsorption, and potential instability [24,25]. Consequently, potentiometric biosensors for blood analysis often require protective or antifouling coatings, microdialysis sampling, or pretreatment membranes to ensure reproducible operation [26].

Urine represents a more accessible and less invasive matrix, frequently used for monitoring metabolic products, drugs, and biomarkers of renal function. Its typically low protein content and high excretion volume are advantageous for sensing; however, large variations in analyte concentration, pH, and ionic composition among individuals and over time complicate calibration [27,28]. Portable potentiometric systems for urinalysis have been explored for rapid point-of-care testing, particularly for detecting electrolytes, glucose, and drug metabolites [29].

Saliva and sweat are gaining increasing attention in the context of wearable and continuous monitoring platforms [30,31,32,33]. Sweat has been used as a proxy for electrolyte and metabolite balance, and several recent wearable potentiometric sensors have demonstrated continuous measurement of Na^+^, K^+^, and lactate during physical activity [34]. Saliva has emerged as a potential diagnostic medium for stress hormones (e.g., cortisol), therapeutic drug levels, and inflammatory biomarkers [35].

Emerging interest is also directed toward interstitial fluid (ISF), which closely mirrors blood composition and can be sampled via minimally invasive microneedle or reverse iontophoresis systems. ISF is especially promising for continuous biosensing applications, as demonstrated by wearable glucose sensors that have inspired similar potentiometric designs for small-molecule monitoring [36,37].

Overall, understanding the physicochemical characteristics and biological variability of each fluid is crucial for the rational design of potentiometric biosensors. The selected medium dictates not only the required detection sensitivity and selectivity, but also membrane composition, transducer material, and calibration approach necessary to achieve reliable and clinically relevant performance.

Table 1 summarizes the main characteristics of commonly used biological fluids, blood/plasma, urine, saliva, sweat, and interstitial fluid, highlighting their representative analytes, typical concentration ranges, and the key benefits and challenges associated with their use in small-molecule potentiometric biosensing.

In conclusion, the wide diversity of clinically relevant small molecules underscores the need for adaptable recognition and transduction strategies. Their distribution across biological fluids shapes the analytical requirements and ultimately influences biosensor performance.

## 4. Materials and Design Strategies for Potentiometric Biosensors

The success of potentiometric biosensors for detecting small molecules in biological fluids lies not only in the sensing chemistry, but critically in how materials are engineered and integrated into sensor architecture. In this chapter, we explore design strategies and material choices: ion-selective membranes and recognition layers, biorecognition element integration, and substrate and format considerations (miniaturization, wearables, paper).

### 4.1. Ion-Selective Membranes as Functional Recognition Interfaces

Recent developments have redefined the ion-selective membrane (ISM) from a passive ionic barrier into an active, multifunctional recognition interface tailored for small-molecule potentiometric biosensing. Contemporary ISMs now combine selective molecular recognition, controlled ion transport and robust mechanical properties to operate directly in biological fluids [6]. Hybrid polymeric matrices, including organically modified sol–gel networks, polyurethane and hydrogel systems, enable encapsulation and stable immobilization of biorecognition elements (enzymes, aptamers, MIPs) while preserving ionic mobility and transduction efficiency [38].

Nanofiller incorporation (carbon nanomaterials, metal oxide nanoparticles, and porous inorganic fillers) improves membrane conductivity, increases surface area for receptor loading, and reduces leaching of lipophilic components [12]. Composite membranes that combine conducting polymers or ionic liquids with conventional PVC/plasticizer matrices can also enhance ion-to-electron coupling and lower detection limits for neutral small molecules when indirect ionic transduction is employed.

Surface and interfacial engineering are equally important. Hydrophobic solid contacts and membrane surface modifications mitigate aqueous film formation at the SC/ISM interface, a major source of potential drift, and promote stable long-term operation [6,12]. To operate reliably in proteinaceous fluids (serum, plasma, saliva), membranes increasingly adopt antifouling strategies such as polyethylene glycol grafting, zwitterionic coatings, or ultrathin permselective layers that reduce nonspecific adsorption while maintaining analyte permeability [39].

Embedding molecularly imprinted polymers directly within or on top of the ISMs provides a robust route for selective recognition of neutral or weakly ionic small molecules (e.g., neurotransmitters, drugs) and has been shown to deliver useful selectivity in complex samples when combined with optimized membrane porosity and surface chemistry [40]. Finally, non-covalent aptamer-membrane interfacing enable sensitive and reversible binding of small organic targets with preserved sensor responsiveness in biological environments [41].

Collectively, these advances demonstrate that modern ISMs are multifunctional platforms where membrane composition, nanostructuring, antifouling and receptor integration must be co-optimized to achieve reliable potentiometric detection of small organic molecules in biological fluids. Advanced ion-selective membranes in potentiometric biosensors are moving toward multifunctional, nanostructured, and biointegrated interfaces.

### 4.2. Solid-Contact Transducer Materials

In SC-ISEs, the ion-to-electron transduction mechanism typically relies on either redox capacitance (in conducting polymers such as polypyrrole, PEDOT:PSS) or double-layer capacitance (in carbon nanomaterials and metallic nanostructures). An efficient solid contact should exhibit high capacitance, chemical inertness, hydrophobicity, and a stable potential even under fluctuating ionic or redox conditions [42]. The prevention of water layer formation between the membrane and the transducer is particularly critical, as it can lead to potential drift and instability over time [43,44] (for detailed discussion on water layer see Section 6).

Material innovations have played a key role in enhancing SC-ISE performance. Conducting polymer–nanocarbon hybrids (e.g., PEDOT:PSS/CNTs, polyaniline/graphene) combine redox and capacitive mechanisms, offering improved charge transfer and minimized noise. Similarly, metal oxide nanoparticles (e.g., RuO_2_, IrO_2_, TiO_2_) and ionic liquids have been explored for their high capacitance and compatibility with biological environments [45]. The use of 3D porous nanostructures and carbon nanofibers can further increase the effective surface area and stability of the transduction interface.

Emerging designs are integrating biorecognition layers directly onto SC-ISEs, forming true potentiometric biosensors. These architectures enable selective detection of small molecules (e.g., neurotransmitters, metabolites, drugs) through enzymatic, aptameric, or molecularly imprinted interfaces. The combination of miniaturized SC-ISEs with flexible substrates, microfabrication, and wireless readout systems is paving the way for wearable and implantable potentiometric biosensors, capable of continuous monitoring in complex biological fluids.

### 4.3. Integration of Biorecognition Elements

#### 4.3.1. Enzymes

**Figure 2:**
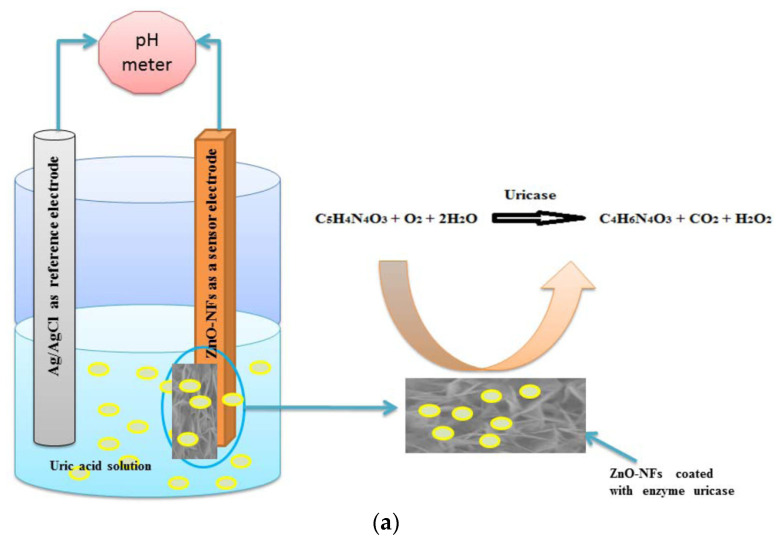

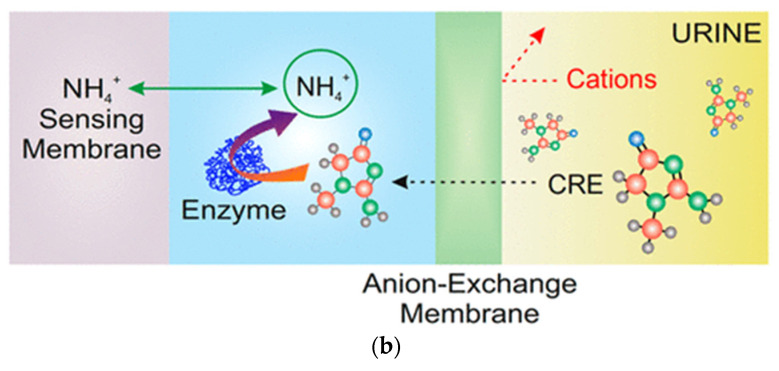
(**a**) Schematic representation of the uric acid sensing setup using ZnO-NFs coated with immobilized uricase form the working electrode, where enzymatic oxidation of uric acid generates ionic products detectable as a potential shift [48]; (**b**) Architecture of a thin-layer enzymatic potentiometric biosensor for creatinine in undiluted urine: creatinine (CRE) diffuses through an anion-exchange membrane, is converted by creatinine deiminase to ammonium ions, and is subsequently quantified by an NH_4_^+^-selective membrane electrode [50].

The use of enzymes in electrochemical biosensors dates back to the pioneering work of Clark and Lyons (1962), who first proposed the concept of an enzyme-based electrode for glucose determination [46]. Enzymes act as catalytic recognition units, transforming analytes into electroactive or ionic products that can be measured potentiometrically through changes in potential across an ion-selective membrane or a solid-contact transducer. The resulting potential variation typically arises from pH changes or the generation of charged species (e.g., H^+^, NH_4_^+^), making enzymatic catalysis a natural bridge between chemical specificity and ionic transduction. In early pH-based potentiometric enzyme electrodes [47], the enzymatic conversion of glucose or urea led to detectable potential shifts correlated with analyte concentration. Subsequent developments incorporated immobilized urease or uricase enzymes within sol–gel or polymeric matrices, enabling stable and selective potentiometric sensing in biological samples. For instance, Ali et al. reported an indirect potentiometric uric acid biosensor using uricase immobilized on ZnO nanoflakes, demonstrating selective detection of uric acid in serum and saliva with excellent sensitivity [48]. The sensing mechanism of electrochemical uric acid sensors is based on an enzymatic reaction catalyzed by uricase as described in Figure 2a. Similarly, potentiometric biosensors employing urease have been designed for non-invasive urea monitoring, including flexible patches for sweat analysis, where the enzymatic hydrolysis of urea generates measurable ammonium ions [49].

Enzymatic potentiometric biosensors often rely on biocatalytic conversion of nonionic or weakly ionic analytes into electroactive ionic species that can be detected by ion-selective membranes. A representative example is illustrated in Figure 2b, showing a thin-layer potentiometric biosensor for creatinine (CRE) detection in undiluted urine demonstrated by Liu et al. [50]. In this configuration, CRE diffuses through an anion-exchange membrane (AEM) that acts as a selective barrier, preventing the passage of interfering cations such as Na^+^ or K^+^. The enzyme creatinine deiminase, confined in an adjacent microfluidic compartment, catalyzes the conversion of CRE into ammonium ions (NH_4_^+^), which are subsequently quantified by an NH_4_^+^-selective membrane electrode. The schematic highlights the diffusion–reaction–detection sequence that underpins this design. The AEM not only enhances selectivity by excluding charged interferences but also enables direct analysis of complex matrices (such as urine) without pretreatment. The device exhibited a Nernstian response with a linear range from 1 to 50 mM, demonstrating how enzymatic potentiometric architectures can achieve clinically relevant small-molecule detection through controlled conversion and ion sensing within integrated microfluidic systems.

Such devices illustrate how enzyme immobilization, trough covalent attachment, entrapment in sol–gel, or adsorption on nanostructured supports, enables high selectivity, fast response, and compatibility with wearable or microfluidic architectures. Nonetheless, enzymatic systems face intrinsic challenges such as limited stability, possible denaturation, and interference from other enzymatic or redox-active species in biological fluids [47,48,49].

#### 4.3.2. Aptamers

While enzymes provide catalytic amplification, aptamers offer a purely molecular recognition route based on conformational changes rather than catalysis. Discovered independently by Tuerk and Gold and Ellington and Szostak in 1990 through the SELEX process [51,52,53], aptamers are short single-stranded DNA or RNA sequences that fold into defined three-dimensional structures capable of selectively binding specific targets, from ions and small molecules to proteins. In potentiometric sensors, aptamer–target binding induces local rearrangements of charge density or steric hindrance at the electrode–electrolyte interface, producing measurable potential shifts without the need for enzymatic conversion.

**Figure 3:**
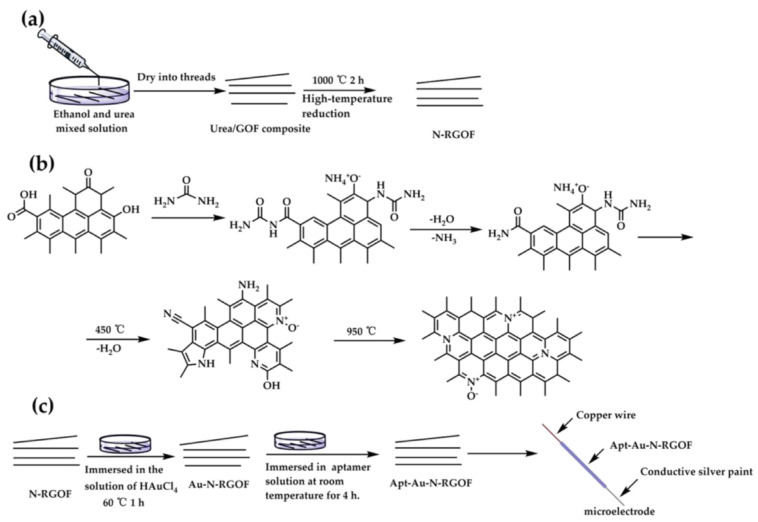
Preparation of N-doped reduced graphene fiber and microelectrode: (**a**) fiber prepared by wet spinning method; (**b**) nitrogen doping mechanism of urea; (**c**) preparation of microelectrode [56].

Early implementations included aptamer-based potentiometric sandwich assays for proteins [53] and label-free thrombin sensors that demonstrated the capacity for reagentless detection through dual-aptamer architectures optimized to minimize nonspecific adsorption [54]. A major advance came with the development of aptamer-based galvanic redox potentiometry (GRP), where aptamer binding modulates redox equilibria at a microelectrode. Using this approach, Ni et al. [55] achieved in vivo detection of dopamine in the rat brain, employing phosphorothioate-modified aptamers that provided high neurocompatibility and selectivity. Similar principles were applied in the graphene microelectrode platform by Zhang et al. (2024), where a surface-tethered dopamine aptamer produced reproducible potential responses in serum and artificial sweat samples [56]. The preparation of microelectrode is shown in Figure 3.

The robustness of such systems relies strongly on chemical stabilization, through 2′-O-methyl or phosphorothioate modifications, and antifouling coatings such as polyethylene glycol or zwitterionic layers to protect the aptamer surface from biofouling in high-ionic-strength matrices [39]. Although aptamer potentiometry is a relatively young field, its rapid progress has positioned it as a promising approach for real-time, label-free, and reversible detection of small organic analytes.

#### 4.3.3. Molecularly Imprinted Polymers (MIPs)

In the meantime, molecularly imprinted polymers (MIPs) have emerged as highly stable and synthetic alternatives to biological receptors. The principle of molecular imprinting was first demonstrated by Wulff and Sarhan in 1972 through covalent template–monomer interactions that left complementary cavities after polymerization and template removal [57]. Later, Mosbach advanced the non-covalent imprinting approach, enabling a wider range of analyte–template interactions and easier polymer synthesis [58,59]. MIPs can mimic the selective binding of antibodies or enzymes but are far more robust, cost-effective, and chemically versatile. In potentiometric biosensors, MIPs act as artificial ionophores or recognition matrices within the sensing membrane, modulating ion partitioning or surface potential upon analyte binding.

**Figure 4:**
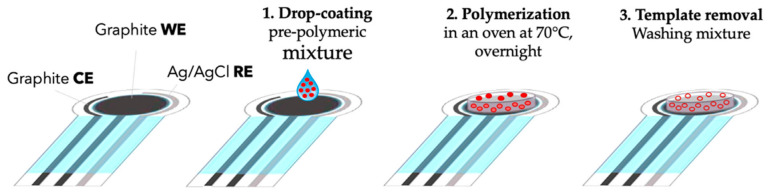
Step-by-step fabrication of a screen-printed potentiometric electrode modified with a molecularly imprinted polymer (MIP). A pre-polymeric mixture containing the template is drop-cast onto the graphite working electrode (WE), thermally polymerized, and subsequently washed to remove the template, creating selective recognition cavities for phenoxy herbicides. The electrode contains also reference Ag/AgCl electrode (RE) and counter graphite electrode (CE) [62].

For instance, Wang et al. [60] reported a MIP-based potentiometric sensor for dopamine using a covalent recognition strategy that achieved a detection limit of 2.1 μM and excellent selectivity in biological media. Zhang et al. [61] later introduced soluble MIPs (s-MIPs) incorporated into polymeric membranes to enhance the uniformity and sensitivity of potentiometric response. Disposable and screen-printed formats have also been successfully demonstrated. For example, Zanoni et al. [62] developed a MIP-modified screen-printed potentiometric device for phenoxy herbicides, highlighting the adaptability of these recognition materials to both environmental and clinical sensing applications. As shown in Figure 4, the fabrication procedure involves drop-coating of a pre-polymeric mixture onto the working electrode, followed by polymerization and subsequent template removal to generate selective binding sites on the sensor surface. More recent work from Kamel et al. [17] has explored stimulus-responsive MIP-based electrodes for small therapeutic drugs such as phenobarbital, integrating the MIP recognition layer with a solid-contact transducer and demonstrating reliable detection in plasma. These examples underline the growing importance of synthetic receptors as durable, reusable interfaces for small-molecule potentiometry.

One of the earliest demonstrations was reported by Moret et al. [63], who developed a potentiometric MIP sensor for carnitine detection in urine samples. The system achieved a limit of detection (LOD) of 80 μM, highlighting the potential of imprinting methods for analytes with moderate polarity. Similarly, Moreira et al. [64] fabricated an MIP-based potentiometric sensor for myoglobin determination in buffer solutions, which showed a linear response above a concentration of 8.0 × 10^−7^ mol/L, confirming the ability of MIPs to detect biologically relevant proteins with good reproducibility. Further extending the applicability to pharmaceutical analysis, Al-Mustafa et al. [65] designed an MIP-modified electrode for Dextromethorphan hydrobromide detection in cough syrup samples, achieving an impressive LOD of 6 × 10^−5^ M and a linear range between 0.01 and 5 × 10^−6^ M. This result demonstrated that MIP-based potentiometric sensors can function effectively even in complex drug formulations. In the biomedical field, Bangaleh et al. [66] proposed an MIP electrode for phenylalanine monitoring in blood serum, reaching a detection limit of 5 × 10^−9^ M and a linear range from 10^−8^ to 10^−4^ M, showing strong potential for use in metabolic disorder diagnostics. The detection of clarithromycin, an antibiotic, in buffer solution was achieved by Radi et al. [67]. Similarly, Mirzajani and Arefiyan in 2019 developed a potentiometric MIP sensor for Dipyridamole in urine and pharmaceutical samples, with a linearity between 2.5 × 10^−8^ and 1.1 × 10^−2^ M, underlining the adaptability of MIP sensors in drug monitoring [68]. Environmental and pharmacological applications have also been demonstrated. SM Hassan et al. [69] reported an MIP-based potentiometric device for dimethylamine quantification, while Babanejad et al. [70] successfully applied the same approach to clonazepam detection, achieving an LOD of 7.3 × 10^−7^ M and a linear range from 10^−7^ to 10^−1^ M. These examples confirm the versatility of MIPs in both environmental and pharmaceutical contexts. Moreover, Sheydaei et al. [71] focused on sarcosine, a potential prostate cancer biomarker, developing a potentiometric MIP biosensor for urine samples with an LOD of 0.38 μM and a linear range of 5.0 μM–1.1 mM, demonstrating its promise for noninvasive cancer diagnostics.

#### 4.3.4. Integration of Elements

Integration of these recognition elements within potentiometric architectures depends critically on immobilization chemistry and membrane engineering. Enzymes are typically entrapped in sol–gel matrices, Nafion, or crosslinked polymers to maintain activity and prevent leaching [47,49,72]. Aptamers are often anchored onto gold or carbon surfaces via thiol or carbodiimide chemistry, with stabilizing modifications to withstand nuclease degradation [55,56,61]. MIPs, in turn, can be synthesized directly on the electrode surface, embedded into PVC/plasticized membranes, or printed as nanoparticle dispersions [60,61,62]. Regardless of receptor type, the interface must balance accessibility to the analyte with protection against fouling; thus, antifouling layers such as polyethylene glycol, zwitterionic polymers, or thin permselective coatings are increasingly used to ensure reliable performance in biological matrices [39].

Ultimately, each recognition strategy offers distinct advantages. Enzyme-based sensors provide catalytic amplification and fast response but require careful stabilization. Aptamer-based sensors deliver reversible and highly specific binding with tunable chemistry, suitable for continuous monitoring applications. MIP-based systems, in contrast, offer unmatched robustness, low cost, and versatility for a broad spectrum of small organic molecules. Ongoing research continues to combine these approaches—for example, hybrid membranes embedding MIPs and aptamers or co-immobilizing enzymes with synthetic receptors—to harness synergistic recognition and improve signal stability. Such integration represents the next step toward reliable, selective, and miniaturized potentiometric biosensors capable of real-time small-molecule monitoring in complex biological environments.

### 4.4. Substrate and Format Choices

In the practical application of potentiometric biosensors for small-organic-molecule detection, the selection of substrate and device format is crucial. It defines the sensor’s analytical robustness, mechanical compliance, and integration capability with electronic and sampling modules. Current progress has followed three main directions: miniaturized microfabricated systems, flexible or wearable platforms, and disposable paper-based devices. These strategies collectively move potentiometric transduction toward real-time, point-of-care chemical monitoring of metabolites, drugs, and neurotransmitters in biological fluids.

#### 4.4.1. Miniaturized and Microfabricated Platforms

Microfabrication enables the construction of miniaturized potentiometric devices capable of handling microliter-scale samples while maintaining high signal sensitivity. For small organic molecules, this architecture allows coupling of selective recognition membranes with stable solid-contact layers and high-throughput readout electronics.

**Figure 5:**
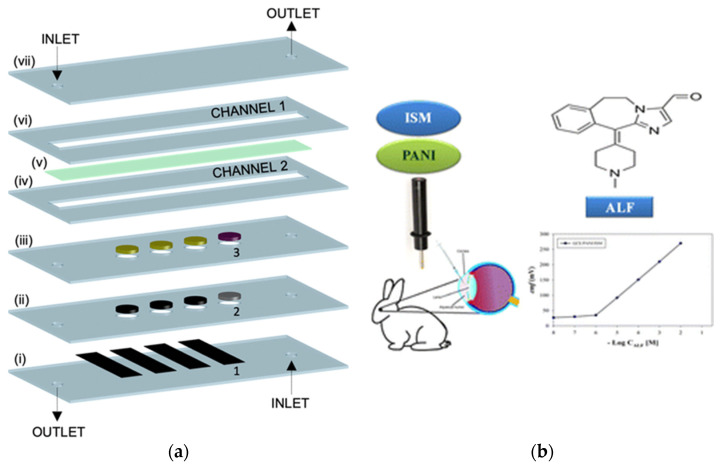
Schematic representation of miniaturized platforms: (**a**) layer-by-layer construction of a microfluidic thin-layer potentiometric biosensor for creatinine, including stacked microchannels, ion-selective membranes, and an enzyme reservoir enabling direct measurement in undiluted urine [50]; (**b**) solid-contact potentiometric sensor for the antihistamine alcaftadine (ALF), incorporating a polyaniline (PANI) transducer layer on a glassy-carbon electrode to enhance potential stability in biological matrices [20].

Miniaturized and microfabricated potentiometric biosensors often rely on multilayered lab-on-a-chip (LOC) architectures that integrate ion-selective electrodes, enzyme layers, and microfluidic channels within compact polymeric or glass substrates. Such configurations allow precise control of sample flow, enhanced mass transport, and the possibility of multiplexed detection in very small sample volumes. For example, the thin-layer potentiometric creatinine biosensor developed by Liu et al. [50] in 2020 demonstrated a multilayer microfluidic assembly incorporating anion-exchange membranes and enzyme reservoirs, achieving analytical performance compatible with clinical assays in undiluted urine. The schematic in Figure 5a illustrates the assembly sequence of the LOC platform, highlighting the integration of microchannels, sensing membranes, and electrode layers within a modular chip design [50].

Miniaturized potentiometric sensors increasingly exploit nanostructured and conducting polymer interfaces to improve signal stability and enable in situ drug monitoring in biological systems. Hussein et al. (2023) fabricated a solid-contact potentiometric electrode for the antihistamine drug alcaftadine (ALF), integrating a polyaniline transducer on glassy carbon; the sensor exhibited excellent reproducibility and potential stability in biological fluids [20]. The device successfully quantified ALF in rabbit body, illustrating its potential for real-time ophthalmic drug monitoring. As shown in Figure 5b, the combination of PANI-based solid contact and ISM architecture demonstrates how compact, biocompatible electrodes can bridge pharmaceutical sensing and clinical microanalysis in miniaturized formats.

Similarly, Ferreira et al. (2021) employed a supramolecular receptor—cucurbituril [6]—embedded in a polymeric membrane for the potentiometric determination of atropine, demonstrating how engineered hosts can act as selective binding sites for small organics [73].

These examples underline how miniaturization and surface nanostructuring improve analyte transport and electrode reproducibility while enabling multiplexed drug and biomarker analysis in compact chip-like systems.

#### 4.4.2. Flexible and Wearable Potentiometric Biosensors

Flexible substrates, such as polyimide (PI), polyethylene terephthalate (PET), polydimethylsiloxane (PDMS) or even textile fibers, permit conformal contact with the human body for non-invasive biosensing in sweat, saliva or interstitial fluid. While wearable potentiometric sensors originally targeted electrolytes, similar architectures now enable the continuous tracking of small organic metabolites and therapeutic drugs.

Ibáñez-Redín et al. (2023) developed a wearable enzymatic potentiometric patch for urea detection in sweat [74]. Urea detection could be performed in the wide range from 5 to 200 mM at pH 7.0, encompassing urea levels in human sweat. This work demonstrated the mechanical feasibility of enzymatic potentiometry on flexible substrates.

Recent advances also demonstrate the feasibility of potentiometric microneedle patches and sweat-ion sensors for on-body chemical monitoring. For instance, in vivo transdermal microneedle patches now enable real-time, multiplexed ion monitoring during continuous wear [75].

Such advances confirm that flexible potentiometric substrates can sustain the immobilization of enzymes, aptamers or MIPs for selective detection of pharmacological analytes, provided that water-layer formation and interfacial drift are controlled.

#### 4.4.3. Paper-Based and Disposable Platforms

Paper-based analytical devices (PADs) have emerged as a powerful class of low-cost and disposable sensing platforms for chemical and biological analysis. Their intrinsic capillarity enables sample transport without external pumps, while cellulose fibers provide a versatile support for surface modification and integration of functional nanomaterials. As a result, PADs have become particularly attractive for portable potentiometric and electrochemical biosensing in point-of-care diagnostics, food safety monitoring, and environmental testing. The inherent biodegradability and printability of paper substrates further facilitate mass production and sustainable single-use operation.

**Figure 6:**
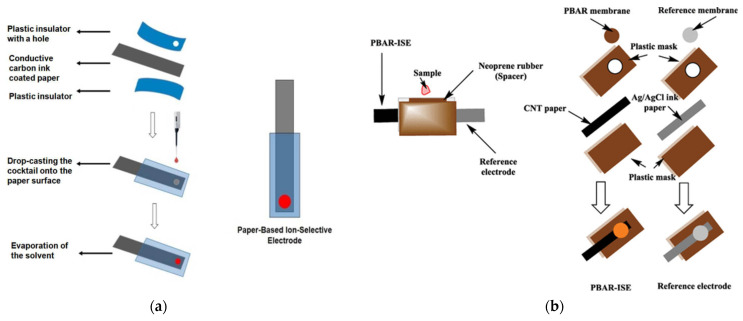
Schematic illustration for fabrication of the potentiometric paper-based sensors: (**a**) ion-selective electrode (ISE). The conductive carbon ink-coated paper is sandwiched between plastic insulators, followed by drop-casting of the ion-selective cocktail and solvent evaporation. Reprinted with permission from RSC Publishing [76]; (**b**) a disposable paper-based potentiometric strip for phenobarbital detection: a CNT paper electrode coated with Ag/AgCl ink and a PVC membrane containing a lipophilic ion-exchanger is combined with a rubber spacer and printed reference electrode, forming a ready-to-use analytical device for drug determination in plasma [7].

Paper-based analytical devices (PADs) have attracted intense interest as low-cost, disposable potentiometric sensors for small organic molecules. Their intrinsic capillarity facilitates sample delivery without external pumps, and printed carbon electrodes enable mass production. In 2018, Kamel et al. [76] proposed a simple and low-cost disposable paper potentiometric sensor based on MIP for the determination of BPA (Bisphenol A). The sensor displayed a linear potentiometric response in the range of 0.5–13 µM, a LOD of 0.15 µM and a good selectivity among other phenols. The schematic representation in Figure 6a illustrates the typical fabrication steps of a paper-based ion-selective electrode (ISE), including the assembly of the conductive paper layer between insulating films, the drop-casting of the ion-selective cocktail, and solvent evaporation leading to sensor activation.

Later, in 2023, the same research group introduced an improved paper-based potentiometric strip for phenobarbital determination. The device incorporated a PVC membrane doped with a lipophilic ion-exchanger and was integrated with a printed reference electrode on CNT paper, allowing fully disposable analysis. As illustrated in Figure 6b, the sensor design relies on a laminated structure with a neoprene spacer to form a sampling reservoir, enabling simple plasma application without external instrumentation. The developed system achieved a detection limit of 0.5 × 10^−7^ M and allowed accurate quantification of phenobarbital in spiked human plasma [7].

Collectively, these studies demonstrate the versatility of paper-based and disposable potentiometric sensors as sustainable analytical tools. Their low cost, rapid response, and compatibility with green fabrication methods make them ideal for point-of-care and field testing. The integration of MIPs, nanomaterials, and microfluidic designs continues to enhance analytical performance, while smartphone-assisted readout and calibration-free operation are paving the way for next-generation wearable and on-site sensing technologies. Such disposable configurations eliminate the need for maintenance, are biodegradable and inexpensive, and are particularly suited for rapid therapeutic drug screening or field diagnostics [77,78].

Miniaturized and microfluidic potentiometric platforms have evolved in parallel with major advances in wearable and paper-based electrochemical systems developed by leading groups in the field. Wang and co-workers pioneered fully integrated wearable and epidermal electrochemical devices including tattoo-based and mouthguard biosensors, that illustrate how microfluidic routing, solid-contact transduction, and real-sample operation can be combined in a single platform [79,80]. Escarpa’s group has demonstrated highly engineered μPAD-based microfluidic architectures for clinical diagnostics, including paper devices capable of analyzing transferrin saturation in patient samples with excellent analytical performance [81]. In addition, seminal work by Arduini et al. has advanced sustainable electrochemical paper-based sensors, showing how controlled electrode printing and eco-designed substrates can dramatically improve device stability and reproducibility [82]. Henry’s group has produced landmark reviews and methodological frameworks for both μPADs and ePADs, establishing the design principles, fabrication strategies, and analytical use-cases that define the state of the art in miniaturized microfluidic platforms [83,84]. Incorporating these developments provides broader context for potentiometric biosensors as part of the expanding landscape of microfabricated electrochemical devices.

### 4.5. Comparative Summary and Discussion

To facilitate direct comparison, Table 2 provides a unified overview of enzymes, aptamers, and MIPs as recognition systems, summarizing key performance parameters including LOD and linear range. The table compiles only those examples for which analytical parameters were explicitly described in the cited works, including the type of recognition element, electrode or membrane composition, device configuration, and characteristic figures of merit such as detection limit and linear range. The selection highlights representative enzyme-, aptamer-, and molecularly imprinted polymer (MIP)-based platforms and illustrates the diversity of design strategies currently explored in the field.

Enzyme-based potentiometric biosensors remain the most established class among the reviewed systems, offering reliable selectivity and well-understood mechanisms of signal generation. Their operation relies on enzymatic catalysis producing ionic species that modulate the interfacial potential, typically yielding near-Nernstian slopes and detection ranges from sub-micromolar to millimolar levels. The reviewed examples demonstrate effective detection of metabolites such as uric acid, urea, and creatinine, often within native biological fluids such as serum, sweat, and urine. Integration with nanostructured ZnO layers, sol–gel matrices, or polymeric supports has enabled their miniaturization into microfluidic and wearable devices, confirming their suitability for real-time and point-of-care use. However, the main drawback of enzyme-based sensors remains their limited operational stability, as enzyme denaturation, pH dependence, and susceptibility to matrix interferences reduce long-term reproducibility. Although immobilization strategies improve retention of enzymatic activity, they often add complexity and cost to device fabrication.

Aptamer-based potentiometric biosensors, in contrast, represent a newer yet rapidly developing approach. These systems exploit the conformational and charge rearrangements of surface-tethered nucleic acid aptamers upon target binding, translating molecular recognition directly into an electrochemical potential shift. Their reported sensitivity, reaching sub-nanomolar levels in complex matrices and even in vivo, underscores their high affinity and reversibility. The studies included in the manuscript demonstrate their successful application for neurotransmitter detection, particularly dopamine, using graphene and nanofiber-based microelectrodes. Despite these promising results, several challenges remain. The performance of aptamer sensors depends strongly on surface chemistry optimization and antifouling strategies to preserve aptamer structure and prevent nonspecific adsorption. Moreover, their fabrication remains less standardized than enzymatic systems, and degradation of nucleic acid receptors in biological media continues to limit their durability.

Molecularly imprinted polymer (MIP)-based sensors dominate the literature quantitatively and exemplify the trend toward low-cost, robust, and disposable platforms. MIPs provide synthetic recognition sites complementary in shape and functionality to their target molecules and are inherently more stable than biological receptors. The reviewed examples cover a broad analyte range, from small metabolites and drugs to biomarkers and environmental pollutants, with detection limits typically between 10^−7^ and 10^−5^ M and linear ranges extending into the millimolar region. Their mechanical robustness and chemical inertness make them particularly attractive for screen-printed and paper-based potentiometric devices, which are well suited for point-of-care applications. Nonetheless, MIPs often suffer from nonspecific binding, slow analyte diffusion into the polymeric matrix, and reduced selectivity for highly polar or hydrophilic molecules. These limitations, while not undermining their potential, highlight the need for continued optimization of imprinting chemistries and transducer interfaces.

Across all biorecognition strategies, design evolution clearly trends toward miniaturized, flexible, and solid-contact architectures that integrate conductive polymers, nanostructured carbons, and metal oxides to enhance charge transfer and mechanical stability. Microfabricated potentiometric chips provide high precision and multiplexing potential, while wearable devices enable continuous, non-invasive monitoring in real time. Paper-based platforms, in turn, offer exceptional affordability and simplicity, positioning them as attractive disposable options for decentralized testing. However, each format faces distinct challenges: microchips require complex fabrication and encapsulation, wearable sensors are prone to mechanical deformation and biofouling, and paper-based systems suffer from limited durability and environmental stability. Despite these differences, the overall direction of development reflects a shared goal of portable, user-friendly potentiometric biosensing. The next generation of devices is likely to emerge from hybrid architectures such as flexible microfluidic systems incorporating disposable paper sampling modules or MIP, and aptamer-based membranes on soft substrates with wireless signal transduction. These integrated approaches hold promise for achieving reliable, real-time detection of therapeutic drugs, metabolites, and neurotransmitters in biological fluids such as sweat, saliva, and interstitial fluid. Yet, fundamental obstacles persist: calibration stability, long-term reproducibility, and antifouling performance continue to limit clinical translation.

Among current approaches, enzyme-based sensors provide the most established analytical performance, aptamer-based devices deliver dynamic and reversible recognition with high specificity, and MIP-based platforms offer robustness and manufacturability.

Taken together, these material and design considerations highlight the interconnected roles of membranes, biorecognition elements, and solid-contact layers. Their thoughtful integration is key to achieving stable, selective, and reproducible potentiometric biosensors.

## 5. Comparison with Other Biosensing Technologies

Potentiometric biosensors occupy a distinct position among electrochemical and physical transduction platforms used for the detection of small molecules in biological fluids. Their working principle, based on measuring the potential difference at an ion-selective or recognition interface, differs fundamentally from that of amperometric, optical, and piezoelectric systems, each of which translates molecular recognition events into different measurable signals. Understanding the advantages and limitations of these approaches is essential for selecting appropriate technologies for biomedical and point-of-care applications.

### 5.1. Comparison with Other Electrochemical Techniques

Amperometric biosensors are historically the most widespread, exemplified by commercial glucose meters that detect enzymatically generated currents proportional to analyte concentration. Their strength lies in high sensitivity, quantitative current readouts, and relatively simple calibration [85,86]. However, amperometric sensors often require external bias potentials, mediators, or redox cofactors, making them susceptible to interference from electroactive species naturally present in biological matrices. In contrast, potentiometric biosensors operate under near-zero current conditions, which minimizes power consumption and prevents electrolysis of the sample. For small-molecule analysis, this property is particularly beneficial in long-term monitoring, as it enables continuous, drift-free operation without reagent depletion or electrode fouling.

Potentiometric biosensors differ fundamentally from other electrochemical techniques such as cyclic voltammetry (CV), electrochemical impedance spectroscopy (EIS), and differential pulse voltammetry (DPV). Unlike voltammetric methods, potentiometry measures the equilibrium potential at or near zero current, enabling low-power operation, compact instrumentation, and a direct, label-free readout [87]. This makes potentiometric platforms especially well-suited for continuous monitoring, wearable sensors, and miniaturized devices [5]. In contrast, CV provides mechanistic insight into redox processes under dynamic potential sweeps but generally requires higher power consumption and cannot easily be sustained for long-term portable monitoring [88]. EIS is very sensitive to interfacial changes—ideal for probing surface modifications and biorecognition events, but mandates more complex instrumentation and model-based data interpretation [87]. Meanwhile, DPV delivers exceptional analytical sensitivity and low detection limits due to its pulsed potential scheme, but it relies on electroactive species or redox labels and tends to suffer more from fouling in complex biological fluids.

### 5.2. Comparison with Other Techniques

Optical biosensors, including fluorescence, surface plasmon resonance (SPR), and colorimetric assays, offer exceptional sensitivity and multiplexing potential. They can achieve picomolar to nanomolar detection limits but usually rely on bulky optics, surface functionalization, or labels, which increase system cost and complexity. For example, SPR-based immunosensors have been applied to drug and metabolite monitoring, yet their integration into wearable or portable platforms remains difficult because of strict alignment and environmental stability requirements [89,90,91]. In contrast, potentiometric devices can be fully miniaturized using screen-printing, flexible substrates, or paper-based formats, enabling direct measurement in microliter-scale samples or even continuous detection in sweat or interstitial fluid. This inherent simplicity positions potentiometric transduction as an attractive alternative for point-of-care (POC) diagnostics.

Piezoelectric biosensors, such as quartz crystal microbalance (QCM) and surface acoustic wave (SAW) systems, rely on measuring frequency shifts resulting from mass loading during analyte binding. They are label-free and capable of real-time analysis, but their performance depends strongly on surface cleanliness and mechanical stability. In viscous or complex biological matrices, damping of acoustic waves limits sensitivity and reproducibility [92,93,94]. Potentiometric systems, conversely, remain functional even in turbid or heterogeneous samples, as the signal arises from interfacial electrochemical potential rather than mass changes.

### 5.3. Discussion

From an analytical performance point of view, potentiometric sensors typically exhibit limits of detection in the micromolar to sub-micromolar range for small molecules, which compares favorably with most label-free amperometric and piezoelectric devices but is generally less sensitive than optical fluorescence assays. However, the selectivity of potentiometric biosensors can be finely tuned by engineering ion-selective membranes, enzyme layers, aptamers, or molecularly imprinted polymers, providing specificity comparable to antibody-based optical platforms. Moreover, since potentiometric sensors measure activity rather than concentration, they inherently reflect the thermodynamic form of an analyte in equilibrium with its matrix, which can be an advantage in physiological monitoring where protein binding affects free analyte levels.

Because potentiometric sensors do not require redox-active analytes and operate under equilibrium or quasi-equilibrium conditions, they avoid many of the limitations associated with current-based methods in biological fluids. Their simple design, tunable selectivity and compatibility with solid-contact architectures provide a unique set of advantages for real-time measurement of ions and small molecules. Nevertheless, current- or impedance-based techniques remain advantageous when detailed mechanistic information, kinetic studies or redox specificity are required. Together, these approaches are complementary, and understanding their differences helps position potentiometric biosensing within the broader electrochemical landscape.

In terms of integration and practical deployment, potentiometric biosensors have demonstrated superior adaptability for wearable and disposable systems. Their solid-contact architectures eliminate the need for internal liquids, enabling flexible and skin-mountable formats that maintain potential stability under motion or bending [19]. Paper-based potentiometric platforms have further simplified fabrication and sample handling, allowing cost-effective, single-use diagnostics without external power sources [7,95,96]. While amperometric and optical sensors dominate high-sensitivity laboratory assays, potentiometric designs excel in low-power, portable, and continuous-monitoring applications where simplicity and robustness outweigh ultimate sensitivity.

## 6. Challenges and Limitations

Despite their numerous advantages, potentiometric biosensors still face several critical challenges that limit their broad adoption in real-world biomedical diagnostics. These challenges are largely associated with the complex composition of biological fluids, the long-term stability of sensing interfaces, and the lack of standardized calibration and data interpretation protocols. When implementing small-molecule detection in biological fluids via potentiometric biosensors, several analytical and operational challenges must be addressed to ensure accurate and reproducible measurements.

### 6.1. Biofouling and Antifouling Strategies in Potentiometric Biosensing

Biofouling and nonspecific adsorption frequently degrade sensor performance over prolonged operation, leading to potential drift and signal attenuation in continuous or wearable monitoring applications [97]. Biofouling is typically caused by the adsorption of proteins, lipids, polysaccharides, and other biomolecules that form an additional interfacial layer that disrupts the physicochemical equilibrium at the membrane or solid-contact interface. This adsorbed layer can introduce charged functional groups or mask active ion-exchange sites, causing local interfacial charge rearrangement that alters the phase boundary potential. As a result, the measured potential may deviate from the expected Nernstian slope, and the apparent sensitivity of the electrode is reduced even when ion activity in the sample remains constant. Furthermore, biofouling can modify the interfacial capacitance of solid-contact electrodes, leading to slow, progressive changes in the transduction mechanism that manifest as potential drift during long-term measurements. These effects are particularly pronounced in undiluted biological samples such as serum, saliva, sweat, and interstitial fluid, where abundant macromolecules continuously interact with the sensor surface.

One of the most persistent obstacles in potentiometric biosensing is therefore the loss of selectivity and stability resulting from surface fouling, especially in long-term in vivo or continuous monitoring applications, such as wearable sensors for sweat or interstitial fluid analysis. To address this challenge, various antifouling strategies have been developed. Common approaches include the application of hydrophilic polymer coatings such as polyethylene glycol (PEG) or zwitterionic polymers, which reduce protein adsorption by forming hydration layers that act as physical and energetic barriers [98]. Hydrophobic protective membranes (e.g., fluorinated polymers or Nafion barriers) can also limit the penetration of large biomolecules while preserving small-ion transport. In addition, enzymatic self-cleaning interfaces have been explored as a means to degrade or prevent the accumulation of adsorbed biomolecules, maintaining long-term transducer integrity [97]. Nanostructured electrode materials, such as graphene derivatives, nanofibers, or hydrophilic polymer brushes, have also shown promise in minimizing fouling through controlled surface chemistry and reduced adhesion sites. Recent studies demonstrate that incorporating these hydrophilic or graphene-based antifouling layers substantially prolongs sensor functionality in biofluids such as serum and plasma [99]. Together, these strategies highlight the importance of interface engineering in mitigating fouling-induced artifacts and ensuring stable, reliable potentiometric sensing performance over extended periods.

### 6.2. Matrix Effects

The matrix effects, including high ionic strength, variable pH, and the presence of surfactants or proteins that adsorb onto sensor surfaces, can alter the interfacial potential, shift the baseline, and compromise selectivity [4]. Biological fluids such as blood, saliva, urine, and sweat contain complex mixtures of electrolytes, proteins, lipids, metabolites, and small ions that interact with the electrode interface in multiple ways. These interactions modify the physicochemical environment surrounding the membrane or solid-contact layer, producing measurable changes in the potentiometric response.

pH fluctuations in biological fluids further complicate potentiometric measurements. Local changes in proton concentration can modify the charge state of membrane components, biorecognition elements, or ion-exchangers, thus altering their binding affinity and transport kinetics. Even small deviations from physiological pH can shift the equilibrium potential, especially for sensors relying on enzyme-driven ion production or pH-responsive transduction layers. This is particularly relevant for urine, where natural pH can range from 4.5 to 8.0, affecting both enzyme activity and membrane ion-exchange behavior.

Variations in ionic strength also play a critical role. Biological fluids exhibit ionic strengths ranging from relatively low (sweat) to very high (blood plasma), which affects the activity coefficients of ions, and influences the mobility of charged species near the membrane interface. Elevated ionic strength may suppress Nernstian response slopes, introduce non-linearities in calibration curves, and promote competitive binding effects when interfering ions outnumber the analyte. These interferences can obscure the analyte signal, particularly at low concentrations.

To counteract these matrix effects, the design of selective membranes and robust recognition layers has become a central focus. Molecularly imprinted polymers (MIPs), aptamer-modified interfaces, and enzyme-based membranes have shown promise in improving selectivity against competing species, yet complete matrix tolerance remains challenging. Recent developments in calibration-free and pseudo-reference-based potentiometric sensors, which rely on intrinsic equilibrium potentials rather than empirical calibration curves, offer a promising route to mitigate matrix-related errors [100,101,102]. Nevertheless, the intrinsic complexity of biological fluids continues to pose significant challenges, underscoring the need for advanced membrane engineering and systematic evaluation of matrix-induced interferences.

### 6.3. Stability of Potentiometric Response

Ensuring long-term stability of the potentiometric response remains one of the major obstacles in translating potentiometric biosensors to reliable routine or continuous monitoring. Stable performance requires control of calibration frequency, minimization of potential drift, robustness of the reference electrode, and proper handling of very small sample volumes in miniaturized or wearable platforms [5]. To address these challenges, modern systems increasingly employ solid-state reference electrodes, drift-compensation algorithms, dual-sensor or self-referencing architectures, and on-site calibration strategies such as internal standards. Sample-conditioning approaches, including filtration, dilution, or matrix matching, further help maintain signal stability under fluctuating sample conditions [103]. Emerging platforms combine nanostructured solid-contact layers, antifouling surface modifications, and microfluidic flow control to support robust continuous operation in complex biological fluids. Still, long-term drift remains a major limitation, as demonstrated in wearable potentiometric patches and solid-contact sweat sensors [104].

A key contributor to signal instability is the formation of a water layer at the interface between the ion-selective membrane and the solid-contact transducer. This thin aqueous film behaves as an uncontrolled ionic reservoir, causing slow redistribution of ions, redox-state fluctuations, hysteresis, and memory effects—especially during concentration changes or shifts in sample matrix composition. The mechanistic origins of this phenomenon are thoroughly described by Shao, Ying and Ping [105], who highlight the interplay among water uptake, ion diffusion, and redox capacitance in driving long-term drift in SC-ISEs. Hydrophilic transducers promote water-layer formation, whereas hydrophobic, high-capacitance materials—such as superhydrophobic PEDOT derivatives (e.g., PEDOT-C14), carbon nanomaterials, and composite layers—greatly suppress water penetration. Their stability is typically verified using the classical “water-layer test,” where stable responses upon ion exchange indicate the absence of interfacial water accumulation.

Current mitigation strategies focus on designing transducer layers with high redox capacitance and pronounced hydrophobicity, incorporating protective or adhesive interlayers, and optimizing membrane–contact integration. Although these advances significantly reduce drift and hysteresis [12,42], achieving multi-week or multi-month stability—essential for implantable, reusable, or long-term wearable devices—remains an open challenge. As a result, simplified recalibration procedures and self-referencing architectures are increasingly explored to enable practical clinical deployment.

In summary, while potentiometric biosensors offer numerous advantages for small-molecule detection, overcoming the challenges of biofouling, matrix interference, stability, and data standardization is vital for their maturation into clinically robust analytical tools. Ongoing research combining material innovation, surface engineering, and intelligent data processing is expected to play a decisive role in transforming potentiometric sensing from laboratory prototypes into reliable, standardized diagnostic technologies.

### 6.4. Wearable and Continuous Sensing: Sweat-Specific Issues

In wearable and continuous-monitoring potentiometric systems, additional challenges arise from zero-current drift, reference electrode stability, and the dynamic properties of sweat as a sensing medium. Unlike laboratory conditions, wearable sensors operate under fluctuating temperature, hydration levels, perspiration composition, and mechanical stress, all of which contribute to slow baseline shifts even at open circuit. Solid-state reference electrodes—often Ag/AgCl-based or printed quasi-references—are particularly sensitive to chloride depletion, sweat evaporation, and contamination from skin-derived species; these instabilities propagate as potential drift in long-term recordings and may compromise calibration validity [97,106]. Moreover, evaporation at the skin–sensor interface concentrates ions over time, artificially increasing measured activities, while episodic contamination (e.g., cosmetics, lotions, or environmental particles) introduces additional unpredictable shifts [107]. To mitigate these issues, current wearable platforms employ ion-permeable solid electrolytes or hydrogel reservoirs to stabilize reference-electrode chloride activity, microfluidic sweat-collection channels to maintain constant volume, and protective membranes that reduce evaporation and fouling [96,97]. Integration of drift-compensation algorithms and intermittent micro-calibration pulses is also increasingly explored to maintain accuracy during prolonged use [106].

## 7. Future Perspectives

Potentiometric biosensors are evolving rapidly, driven by advances in materials science, microfabrication, and data analytics. These developments are redefining the capabilities of electrochemical sensing platforms and expanding their potential applications in personalized healthcare and point-of-care diagnostics. As the field moves forward, three main trends appear most influential: innovative materials and architectures, digital and AI-based data interpretation, and integration into individualized diagnostic ecosystems.

Emerging materials and sensor architectures are at the heart of next-generation potentiometric biosensors. Traditional ion-selective membranes are being replaced or supplemented by nanostructured materials, such as graphene, carbon nanotubes, that offer high surface area, electrical conductivity, and chemical tunability [15]. These nanomaterials not only improve potential stability and signal transduction but also facilitate direct bioreceptor immobilization. Hybrid materials combining conducting polymers with nanocarbons or metal–organic frameworks (MOFs) have shown particular promise for enhancing both sensitivity and selectivity [12]. Furthermore, flexible and stretchable substrates are enabling the integration of potentiometric sensors into wearable formats, including skin patches, textiles, and even microneedle platforms for interstitial fluid analysis [108]. The miniaturization of reference electrodes and the use of all-solid-state architectures are essential for achieving fully integrated, maintenance-free devices suitable for continuous operation.

The coupling of potentiometric sensors with digital and artificial intelligence (AI)-driven data analysis represents another transformative frontier. Potentiometric responses are inherently non-linear and can be influenced by temperature, drift, and sample matrix variations. AI algorithms, such as machine learning regression, principal component analysis, and neural network, are being increasingly used to compensate for these effects and extract meaningful analyte information from complex datasets [109]. Combining multi-sensor arrays (potentiometric electronic tongues) with pattern recognition and chemometric models allows simultaneous detection of multiple analytes, even in unprocessed biological fluids. Moreover, integration with Internet-of-Things (IoT) platforms enables real-time monitoring, cloud-based data storage, and personalized feedback loops, paving the way for connected healthcare systems where potentiometric sensors serve as front-end diagnostic interfaces [110,111].

Finally, the potential for personalized and point-of-care diagnostics underscores the broader societal and clinical relevance of potentiometric biosensing. The portability, low power consumption, and reagent-free operation of these devices make them ideal for decentralized testing environments. Continuous monitoring of electrolytes, metabolites, and therapeutic drugs using minimally invasive or non-invasive potentiometric sensors could provide clinicians and patients with real-time insights into physiological status [112]. In personalized medicine, such continuous biochemical profiling could guide medication dosing, hydration management, and nutritional strategies, enabling truly adaptive healthcare. However, large-scale clinical validation and standardization efforts will be critical to translating these capabilities into approved medical devices.

Looking ahead, advances in materials, signal processing, and personalized health technologies are expected to accelerate the translation of potentiometric biosensors into routine biomedical practice. Their future impact will depend on seamless integration with digital and flexible sensing platforms.

## 8. Conclusions

Potentiometric biosensors have evolved far beyond their origins as basic ion-selective electrodes. Today, they represent a new generation of analytical tools capable of detecting small molecules in complex biological environments with impressive precision. Unlike amperometric or optical systems, potentiometric sensors measure equilibrium potential rather than current or light, which gives them unique advantages: low power demand, label-free detection, and seamless compatibility with miniaturized or flexible formats. Over the past decade, advances in solid-contact materials, nanostructured transducers, and functional interfaces have dramatically enhanced their analytical performance, paving the way for applications in clinical diagnostics, therapeutic drug monitoring, and continuous physiological sensing.

The choice of biorecognition strategy plays a central role in sensor design. For molecules that can be enzymatically converted into ionic products, such as the hydrolysis of urea to ammonium or the oxidation of uric acid, enzyme-based potentiometric biosensors remain the simplest and most effective option. When enzymatic reactions are not suitable, for example, in detecting neutral small molecules or drugs such as dopamine, molecularly imprinted polymers (MIPs) provide a powerful alternative. For applications that demand reversible and highly specific binding, such as real-time or in vivo monitoring, aptamer-based potentiometric sensors have proven particularly promising.

Despite these advances, some challenges remain before potentiometric biosensors can achieve widespread clinical use. Biofouling, signal drift, and matrix interference still limit reliability in long-term operation. Progress in antifouling materials, surface engineering, and calibration-free sensing is essential, as is the development of AI-driven data interpretation to ensure accuracy, standardization, and regulatory compliance.

Looking ahead, the field of potentiometric biosensors is moving rapidly toward integration with flexible electronics, digital data analytics, and personalized medicine. As laboratory prototypes evolve into practical, wearable, or point-of-care devices, potentiometric biosensors are positioned to play a central role in next-generation healthcare.

## Figures and Tables

**Table 1:** Comparison of biological fluids for small-molecule potentiometric biosensing.

Biological Fluid	Typical Analytes (Examples)	Concentration Range	Advantages for Biosensing	Challenges/Limitations
Blood/Plasma	Glucose, uric acid, creatinine, electrolytes, hormones, drugs	µM–mM	Gold standard; directly reflects systemic physiology; broad range of clinically relevant analytes	Invasive sampling; high protein content; fouling and drift; high ionic strength; need for calibration and pretreatment
Urine	Creatinine, urea, uric acid, drugs/metabolites	µM–mM	Non-invasive collection; relatively low protein content; suitable for point-of-care testing	Variable dilution; pH and ionic composition vary; temporal variability; storage instability
Saliva	Cortisol, lactate, glucose, drugs	nM–µM	Non-invasive, easy sampling; potential for wearable or home monitoring	Low analyte concentrations; secretion rate variability; contamination (food, oral bacteria); temperature effects
Sweat	Lactate, glucose, small metabolites	µM–mM	Real-time, continuous monitoring via wearables; reflects metabolic changes during activity	Low volume; evaporation; contamination; lag time vs. blood concentration; variable secretion
Interstitial Fluid (ISF)	Glucose, lactate, small drugs	µM–mM	Composition close to plasma; accessible via minimally invasive methods (microneedles, iontophoresis); continuous monitoring potential	Requires specialized extraction; slow equilibration; limited standardization

**Table 2:** Potentiometric biosensors for small-molecule detection with reported analytical parameters, recognition element for target molecule and design of a sensor.

Target/Matrix	Recognition Element	Analytical Parameters	Design	Refs.
Uric acid	Uricase (enzyme)	Linear 500 nM–1.5 mM	Solid-contact enzymatic electrode	[48]
Urea (urine)	Urease (enzyme)	Linear 10^−6^–10^−1^ M	Flexible/wearable patch	[49]
Creatinine (urine)	Creatinine deiminase (enzyme)	Linear 1–50 mM	Thin-layer microfluidic chip	[50]
Dopamine (rat brain, in vivo)	Phosphorothioate aptamer	Sub-nM—nM	Implantable in vivo probe	[55]
Dopamine (serum)	Dopamine aptamer	LOD 0.5 µM; Linear 1–100 µM	Graphene-based flexible microelectrode	[56]
Dopamine	MIP	LOD 2.1 µM	Solid-contact MIP electrode	[60]
Carnitine (urine)	MIP	LOD 80 µM	Potentiometric sensor with MIP as ionophore	[63]
Dextromethorphan hydrobromide	MIP	LOD 6 × 10^−5^ M; Linear 0.01–5 × 10^−6^ M	Coated electrodes of polymers imprinted with dextromethorphan hydrobromide	[65]
Phenylalanine (serum)	MIP	LOD 5 × 10^−9^ M; Linear 10^−8^–10^−4^ M	Potentiometric MIP electrode	[66]
Dipyridamole (urine)	MIP	LOD 10^−8^ M; Linear 2.5 × 10^−8^–1.1 × 10^−2^ M	Modified potentiometric carbon paste electrode	[68]
Clonazepam (biological fluid model)	MIP	LOD 7.3 × 10^−7^ M; Linear 10^−7^–10^−1^ M	Modified potentiometric carbon paste electrode	[70]
Sarcosine (urine)	MIP	LOD 0.38 µM; Linear 5 µM–1.1 mM	MIP-based prostate cancer biomarker sensor	[71]
Urea (sweat)	Urease (enzyme)	Linear 5–200 mM	Wearable enzymatic patch	[74]
Bisphenol A	MIP	LOD 0.15 µM; Linear 0.5–13 µM	Disposable paper sensor	[76]
Phenobarbital (urine)	MIP	LOD 5 × 10^−7^ M Linear 10^−6^–10^−3^ M	Paper-based POC device	[7]

## Data Availability

No new data were created or analyzed in this study. Data sharing is not applicable to this article.

## Abbreviations

The following abbreviations are used in this manuscript:

ISE	Ion-Selective Electrode
ISM	Ion-Selective Membrane
SC-ISE	Solid-Contact Ion-Selective Electrode
PVC	Poly(vinyl chloride)
POC	Point-of-Care
QCM	Quartz Crystal Microbalance
SAW	Surface Acoustic Wave
MIP	Molecularly Imprinted Polymer
NIP	Non-Imprinted Polymer
PEDOT:PSS	Poly(3,4-ethylenedioxythiophene):poly(styrene sulfonate)
CNT	Carbon Nanotube
rGo	Reduced Graphene Oxide
AuNP	Gold Nanoparticle
ZnO	Zinc Oxide
LOC	Lab-on-a-Chip
LOD	Limit of Detection
EIS	Electrochemical Impedance Spectroscopy
SEM	Scanning Electron Microscopy
PANI	Polyaniline
PPy	Polypyrrole
PEG	Poly(ethylene glycol)
PEO	Poly(ethylene oxide)
PS	Polystyrene
PDMS	Polydimethylsiloxane
PHEMA	Poly(2-hydroxyethyl methacrylate)
PMMA	Poly(methyl methacrylate)
FET	Field-Effect Transistor
SPE	Screen-Printed Electrode
DOX	Doxorubicin
PBS	Phosphate-Buffered Saline
AI	Artificial Intelligence
TDM	Therapeutic Drug Monitoring
PBA	Phenylboronic Acid
SC	Solid-Contact
WE	Working Electrode
RE	Reference Electrode
